# Mechanistic insight of interleukin-9 induced osteoclastogenesis

**DOI:** 10.1111/imm.13630

**Published:** 2023-02-15

**Authors:** Sushmita Chakraborty, Jakob Schneider, Dipendra Kumar Mitra, Katharina F. Kubatzky

**Affiliations:** 1 Department of Infectious Diseases, Medical Microbiology and Hygiene, Heidelberg University, Heidelberg, Germany; 2 Department of Transplant Immunology and Immunogenetics, All India Institute of Medical Sciences, New Delhi, India

**Keywords:** effector T cells, interleukin-9, osteoclast, regulatory T cells, signal transduction

## Abstract

Interleukin (IL)-9 is an emerging player in the pathogenesis of various chronic inflammatory diseases including bone disorders like rheumatoid arthritis (RA) and psoriatic arthritis. Recently, IL-9 was shown to enhance the osteoclast formation and their function in RA. However, the mechanisms by which IL-9 influences osteoclastogenesis are not known. Therefore, in this study we aimed to unravel the direct and indirect ways by which IL-9 can influence osteoclast formation. We used mouse bone marrow precursor cells for checking the effect of IL-9 on osteoclast differentiation and its function. Next, IL-9 induced signalling pathway were checked in the process of osteoclastogenesis. T cells play an important role in enhancing osteoclastogenesis in inflammatory conditions. We used splenic T cells to understand the impact of IL-9 on the functions of T effector (Teff) and regulatory T (Treg) cells. Furthermore, the effect of IL-9 mediated modulation of the T cell response on osteoclasts was checked using a coculture model of T cells with osteoclast precursors. We showed that IL-9 enhanced osteoclast formation and its function. We found that IL-9 activates STAT3, P38 MAPK, ERK1/2, NFκB and we hypothesize that it mediates the effect on osteoclastogenesis by accelerating mitochondrial biogenesis. Additionally, IL-9 was observed to facilitate the functions of pro-osteoclastogenic IL-17 producing T cells, but inhibits the function of anti-osteoclastogenic Treg cells. Our observations suggest that IL-9 can influence osteoclastogenesis directly by modulating the signalling cascade in the precursor cells; indirectly by enhancing IL-17 producing T cells and by reducing the functions of Treg cells.

## Introduction

Bone remodelling is a finely orchestrated process involving the interaction between two bone cell types: bone-resorbing osteoclasts and bone-forming osteoblasts. This precisely regulated process of the removal of damaged bones by osteoclasts is tightly coupled with the recruitment of osteoblasts for the formation of new bones in order to maintain the skeletal homeostasis. An imbalance in this process is often observed in various skeletal disorders [1].

Osteoclasts are multi-nucleated giant cells, originating from the haematopoietic precursors of the myeloid lineage [2]. Two osteoblast derived cytokines, which are critical for driving the osteoclast differentiation, are macrophage colony stimulating factor (MCSF) and receptor activator of NFκB ligand (RANKL) [3]. Apart from these cytokines, pro-inflammatory cytokines like Tumour Necrosis Factor (TNF)-α and Interleukin (IL)-1β have been reported to mediate osteoclastogenesis in a RANKL independent manner [4, 5]. Additionally, components from bacteria like lipopolysaccharide or bacterial toxins can also induce osteoclastogenesis in a RANKL independent manner [6, 7]. These factors are potent inducers of osteoclastogenesis and can skew the bone remodelling towards bone loss. This is evident from clinical observations, where patients suffering from chronic infections or inflammatory diseases display bone loss [8, 9].

In chronic inflammation, T cells play a critical role in linking inflammation to osteoclastogenesis as activated T cells express RANKL [10]. Additionally, IL-17 producing T cell subsets are potent inducers of osteoclastogenesis and are often enhanced in inflammatory bone disorders [11]. Another T cell subset, regulatory T (Treg) cells which are critical in mediating immune suppression and in inhibition of osteoclastogenesis are often found either reduced or dysfunctional in chronic inflammatory conditions [12]. Together, these phenomena in systemic inflammatory conditions trigger severe bone destruction.

In recent years, IL-9, a member of γc family, has been observed to be involved in the pathogenesis of various chronic inflammatory bone disorders like rheumatoid arthritis (RA), psoriatic arthritis, etc [13]. The cytokine mediates the effect through its heterotypic receptor complex consisting of the IL-9 receptor α (IL-9Rα) and common gamma chain subunit (γc) [14]. Apart from its role as a T cell growth factor, reports indicate its function on various other cell types [15, 16]. Very recently, we have shown that IL-9 facilitates differentiation of human myeloid cells into osteoclasts in RA, an inflammatory bone disease [17]. Therefore, it is important to unravel the mechanism by which IL-9 influences osteoclastogenesis and its bone resorptive function. As T cells play a critical role in shaping the outcome of osteoclastogenesis, we checked the effect of IL-9 on T effector (Teff) and Treg cells in order to understand their influence on osteoclastogenesis.

In this study, we showed that IL-9 has the potential to enhance RANKL induced osteoclastogenesis in mouse bone marrow cells (BMCs), bone marrow derived macrophages (BMDMs) and monocytes. We further elucidated the mechanism by which IL-9 augments RANKL mediated osteoclastogenesis. Besides its direct effect on the differentiation of myeloid cells into osteoclasts, it can influence osteoclastogenesis through modulating the functions of Teff and Treg cells. We showed that it enhances proosteoclastogenic IL-17 producing T cells on the one hand and reduces the function of anti-osteoclastogenic Treg cells on the other hand. Together, our study provides a mechanistic insight by which IL-9 can possibly influence bone remodelling in inflammatory conditions.

## Methods

### Mice

C57BL/6 wild-type mice were purchased from Janvier Labs (Le Genest St. Isle, France). Mice were maintained under SPF conditions in accordance with the German policies on animal welfare.

### Isolation of bone marrow cells, monocytes and bone marrow-derived macrophages

Bone marrow cells (BMCs) were isolated from the femur, tibia and humerus of 6–12 weeks old C57BL/6 mice. Monocytes were isolated as mentioned in Supplementary Methods. For generating bone marrow derived macrophages (BMDM), BMCs were cultured using L929-cell conditioned medium (LCCM) for 6 days as mentioned previously [7].

### RAW 264.7

RAW 264.7 cells (ATCC) were cultured in DMEM.

### Isolation of splenocytes and T cells

Splenocytes; CD4^+^ CD25^−^ (T effector) and CD4^+^ CD25^+^ (T regulatory) cells were isolated as mentioned in Supplementary Methods.

### Real time quantitative polymerase chain reaction

RNA was isolated from cells followed by cDNA synthesis as mentioned in Supplementary Methods. Real-time PCR analysis was performed using primer pairs used as listed in Table S1.

### TRAP assay

TRAP assay was performed using Acid Phosphatase, Leukocyte (TRAP) Kit (Sigma, St. Louis, MO, USA) as mentioned in Supplementary Methods.

### Bone resorption assay

2 × 10^5^ cells were cultivated on bovine cortical bone slices (http://Boneslices.com, Jelling, Denmark). For measurement of resorbed bone, bone slices were washed and stained with 0.1% toluidine blue (Sigma–Aldrich, MO, USA) as mentioned in Supplementary Methods.

### Flow cytometry (FC)

For intracellular cytokines analysis, 0.5 × 10^6^ cells were cultured in vitro in the presence of Dynabeads mouse T cells activator CD3/CD28 (Thermo Fisher Scientific, MA, USA) and supplemented with cytokines IL-2 or IL-9 (PeproTech, NJ, USA) for indicated durations followed by addition of Brefeldin A solution (Biolegend, San Diego, CA, USA) for last 12 h of culture. Following cell culture, cells were proceeded for surface and intracellular staining using antibodies listed in Table S2 as mentioned in Supplementary Methods.

### Immunoblotting

Stimulated cells were lysed using a lysis buffer and immunoblotting was performed as mentioned in Supplementary Methods using antibodies as listed in Table S2.

### Statistical analysis

All data presented as mean ± SD. Statistical analyses were carried out using the GraphPad Prism 9.0 software; *p* ≤ 0.05 was considered statistically significant.

## Results

### IL-9 augments the RANKL mediated osteoclastogenesis

Recently, in RA, we showed that IL-9 enhances the differentiation of human monocytes into osteoclasts [17]. However, the mechanism by which it enhances the osteoclast differentiation is not yet unravelled. In this study, we first established whether the effect of IL-9 we observed in the differentiation of human myeloid cells into osteoclasts is also preserved in mouse myeloid cells. Thus, we assessed the effect of IL-9 on the differentiation process of osteoclasts from bone marrow cells (BMCs).

**Figure 1:**
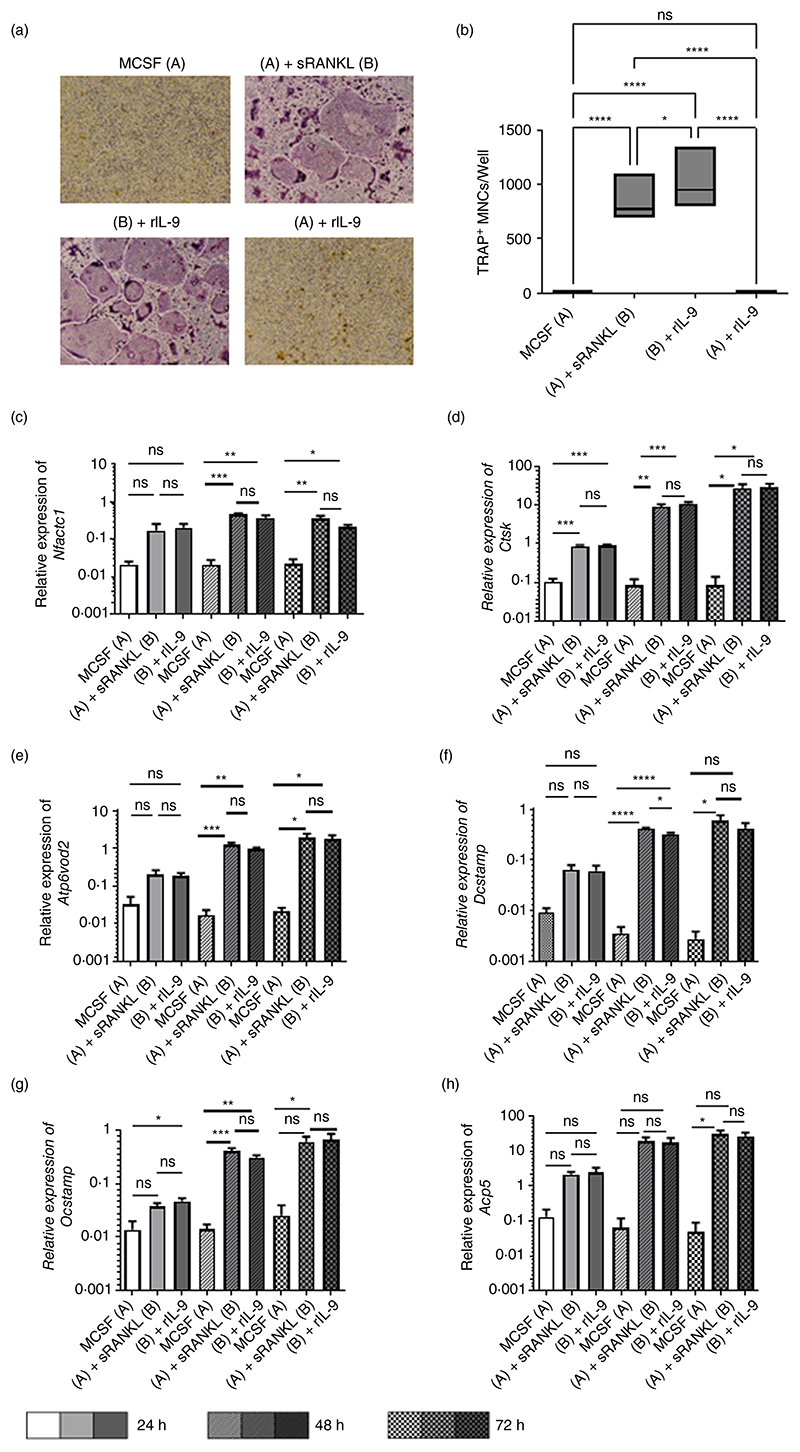
Synergistic effect of IL-9 and RANKL on osteoclast differentiation from bone marrow cells. (a, b) Bone marrow cells (BMCs) were treated as indicated with macrophage colony-stimulating factor (MCSF; 30 ng/mL), soluble receptor activator of nuclear factor κB ligand (sRANKL; 10 ng/mL) or rIL-9 (50 ng/mL) for 5–6 days. After 72 h intervals, half of the culture medium was replenished with fresh culture medium containing stimulating factors (MCSF, sRANKL and rIL-9). Cells were then fixed and stained for tartrate-resistant acid phosphatase (TRAP). Using a light microscope, multinucleated (≥3 nuclei) TRAP^+^ cells were counted manually. (a) Representative picture of TRAP^+^ multinucleated cells (MNCs). (b) Graph showing TRAP^+^ MNCs (mean ± SD; *n* = 7). (c–h) 2 × 10^6^ BMCs were stimulated as indicated with MCSF (30 ng/mL), sRANKL (10 ng/mL) or rIL-9 (50 ng/mL) for 24 h, 48 h and 72 h. Quantitative real-time PCR (RT-PCR) was performed for osteoclast-specific marker genes, nuclear factor of activated T cells, cytoplasmic 1 (*Nfatc1*), cathepsin K (*Ctsk*), d2 isoform of vacuolar (H^+^) ATPase (v-ATPase) V0 domain (*Atp6v0d2*), Dendritic cell-specific transmembrane protein (*Dcstamp*), osteoclast stimulatory transmembrane protein (*Ocstamp*) and acid phosphatase 5, tartrate-resistant (*Acp5*). The graphs represent the relative expression (mean ± SD; *n* = 3). Statistical analysis was performed using one-way ANOVA for multiple comparison (*p ≤ 0.05; **p ≤ 0.005; ***p ≤ 0.0005; ****p ≤ 0.0001; ns, non-significant).

In vitro differentiations of BMCs into osteoclasts require stimulation with MCSF and soluble (s) RANKL. Thus, BMCs were cultured with various combinations of MCSF, sRANKL and recombinant (r) IL-9. MCSF stimulated cells acted as a negative control. Osteoclastogenesis has been shown to be induced in a RANKL independent manner [4–7]. So, we also stimulated BMCs with MCSF and rIL-9 in order to check whether IL-9 has the potential to induce osteoclastogenesis without sRANKL stimulation. Tartrate-resistant acid phoshatase (TRAP)-positive cells with three or more nuclei were scored as osteoclasts. We observed a significant increase in the TRAP^+^ multinucleated osteoclast formation in rIL-9 treated cells along with MCSF/sRANKL compared to the MCSF/sRANKL stimulated cells (Figure 1a,b). IL-9 stimulation along with MCSF did not induce osteoclast formation indicating that IL-9 has the potential to induce osteoclastogenesis in the presence of sRANKL.

We next investigated the expression of osteoclast markers in the cells treated as mentioned above (Figure 1c–h). The analysis of the expression of osteoclast markers revealed that stimulation with MCSF and sRANKL significantly enhanced the expression of nuclear factor of activated T cells, cytoplasmic 1 (*Nfatc1*), cathepsin K (*Ctsk*), d2 isoform of vacuolar (H^+^) ATPase (v-ATPase) V0 domain (*Atp6v0d2*), Dendritic cell-specific transmembrane protein (*Dcstamp*), osteoclast stimulatory transmembrane protein (*Ocstamp*) and acid phosphatase 5, tartrate-resistant (*Acp5*). IL-9 stimulation along with MCSF/sRANKL also significantly enhanced the gene expression of osteoclast markers compared to MCSF stimulated cells. However, we did not observe any significant difference in the gene expression between MCSF/sRANKL treated cells in presence or absence of rIL-9. Thus, understanding the IL-9 mediated signal transduction in monocytes is needed to explain the phenomenon of IL-9 induced effect on osteoclast formation. As BMC are a heterogeneous cell population and not well suited for studying the signal transduction, therefore, it is important to check the effect of IL-9 on the homogenous myeloid population.

### Effect of IL-9 on osteoclast formation from myeloid precursor cells

**Figure 2:**
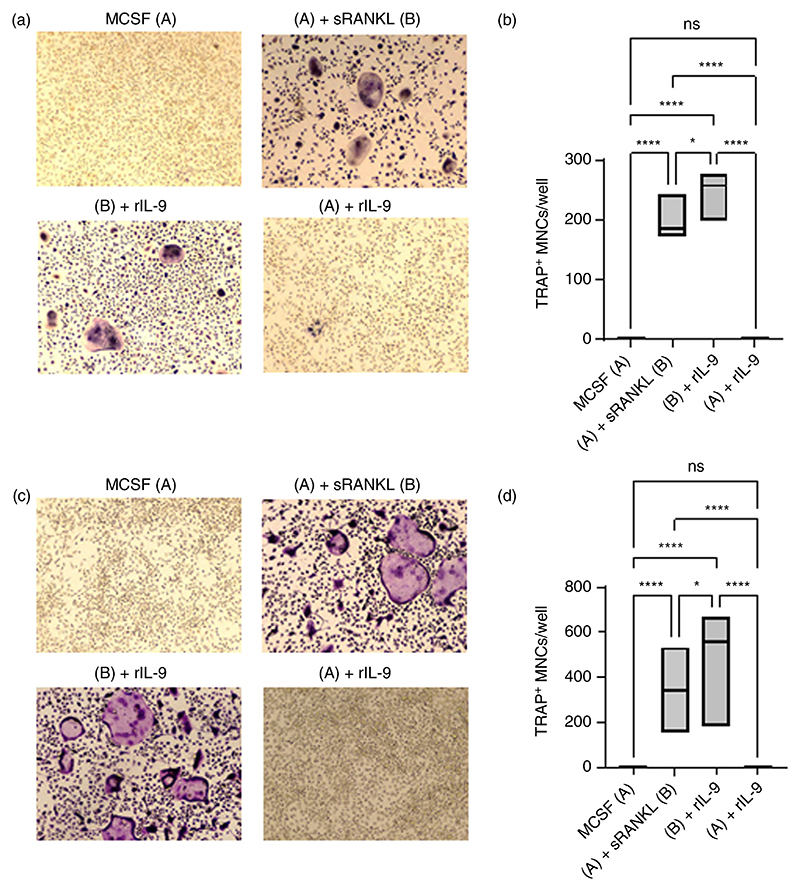
IL-9 enhances osteoclastogenesis in homogeneous populations of monocytes and macrophages. (a, b) 2 × 10^5^ bone marrow derived macrophages (BMDMs) were treated as indicated with macrophage colony-stimulating factor (MCSF; 25 ng/mL), soluble receptor activator of nuclear factor κB ligand (sRANKL; 50 ng/mL) or rIL-9 (50 ng/mL) for 5–6 days. After 72 h intervals, half of the culture medium was replenished with fresh culture medium containing stimulating factors (MCSF, sRANKL and rIL-9). Cells were then fixed and stained for tartrate-resistant acid phosphatase (TRAP). Using a light microscope, multinucleated (≥3 nuclei) TRAP^+^ cells were counted manually. (a) Representative picture of TRAP^+^ multinucleated cells (MNCs). (b) Graph shows TRAP^+^ MNCs (mean ± SD; *n* = 4). (c, d) 2 × 10^5^ CD11b^+^ cells were treated as indicated with MCSF (30 ng/mL), sRANKL (10 ng/mL) or IL-9 (50 ng/mL) for 5–6 days. After 72 h intervals, half of the culture medium was replenished with fresh culture medium containing stimulating factors (MCSF, sRANKL and rIL-9). Cells were then fixed and stained for TRAP. (c) Representative picture of TRAP^+^ multinucleated cells (MNCs). (d) Graph showing TRAP^+^ MNCs (mean ± SD; *n* = 7). Statistical analysis was performed using one-way ANOVA for multiple comparison (*p ≤ 0.05; ***p ≤ 0.0005; ****p ≤ 0.0001).

Next, we investigated the effect of IL-9 on the differentiation of a homogenous population of macrophages or monocytes into osteoclasts. We first investigated the impact of IL-9 on the differentiation of bone marrow derived macrophages (BMDM) into osteoclasts. BMDM cells were stimulated with MCSF and sRANKL in presence or absence of rIL-9 (Figure 2a,b). Similar to BMCs, we observed a significant increase in osteoclast formation with rIL-9 treatment along with MCSF and sRANKL. Next, we investigated the effect of IL-9 on the differentiation of bone marrow derived monocytes (CD11b^+^ sorted cells) into osteoclasts. Similar to BMCs and BMDM cells, we observed significantly enhanced osteoclast formation in CD11b^+^ sorted cells with MCSF and sRANKL stimulation along with rIL-9 treatment (Figure 2c,d; Figure S1).

Together, these observations clearly indicate that IL-9 positively impacts RANKL mediated osteoclastogenesis in primary mouse myeloid cells.

### IL-9 accentuates the activation of signalling intermediates essential for osteoclastogenesis

**Figure 3:**
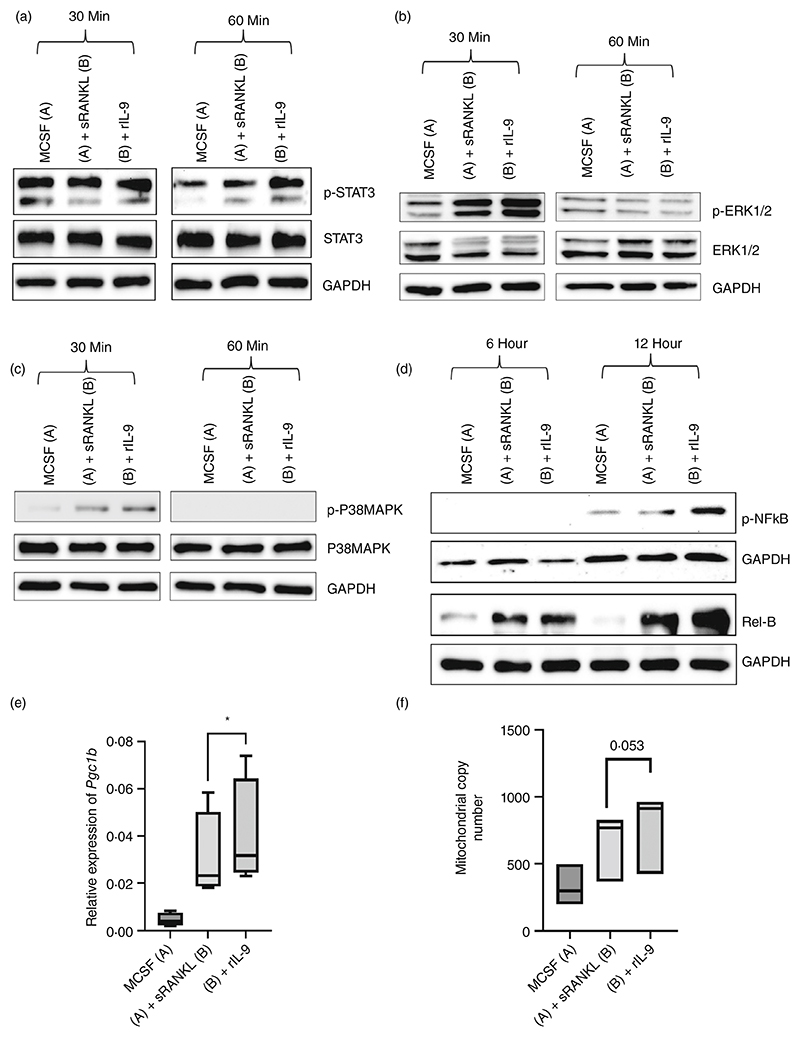
IL-9 mediated activation of signalling intermediates in osteoclastogenesis. (a–d) To investigate the kinetics of IL-9 mediated effect on MCSF/sRANKL induced osteoclastogenesis, BMDM cells were stimulated as mentioned for the indicated time points with macrophage colony-stimulating factor (MCSF; 25 ng/mL), soluble receptor activator of nuclear factor κB ligand (sRANKL; 50 ng/mL) or IL-9 (50 ng/mL). As a control, cells were stimulated with MCSF. (a) The immunoblot was probed with an antibody detecting phosphorylated STAT-3 (pSTAT-3); STAT-3 and GAPDH (*n* = 4). (b) The immunoblot was probed with an antibody detecting phosphorylated ERK-1/2 (pERK-1/2); ERK-1/2 and GAPDH (*n* = 4). (c) The immunoblot was probed with an antibody detecting phosphorylated P38MAPK (pP38MAPK); P38MAPK and GAPDH (*n* = 4). (d) Immunoblot of phosphorylated NFκB (p-NFκB), Rel-B and GAPDH (*n* = 3). (e) Cells were stimulated as indicated for 3 days and expression of *Pgc1b* was investigated by RT-PCR; normalized to the housekeeping gene rsp29 (*n* = 4). (f) Cells were stimulated as indicated for 3 days before preparing genomic DNA. Mitochondrial copy number was determined by RT-PCR comparing the expression of a mitochondria specific sequence with nuclear DNA (*n* = 3). Statistical analysis was performed using paired Student’s *t*-test comparing MCSF/sRANKL/rIL-9-treated cells to MCSF/sRANKL treated cells (*p ≤ 0.05).

IL-9 has been reported to mediate its effect through activation of signal transducer and activator of transcription (STAT)1 or STAT3 or STAT5 [15]. However, RANKL does not mediate its effect through the STAT pathway. Therefore, we checked the activation of STAT1, STAT3 and STAT5 in cells stimulated with indicated stimulus. We observed enhanced phosphorylation of STAT3 after 60 min of stimulation with rIL-9 along with MCSF and sRANKL (Figure 3a). As expected, we did not observe any activation of STAT3 in MCSF and sRANKL stimulated cells. We also checked the phosphorylation of STAT1 and STAT5 in cells stimulated with rIL-9 along with MCSF and sRANKL, but we could not see any activation (data not shown). Yang et al. have mechanistically shown that osteoclast differentiation was impaired in STAT3 deficient macrophages, indicating its importance in osteoclastogenesis [18]. Indeed, we also observed that STAT3 inhibition abrogated the RANKL induced osteoclast formation in the presence or absence of IL-9, indicating its importance in osteoclastogenesis (Figure S2).

IL-9 has been reported to activate extracellular signal-regulated kinase 1/2 (ERK1/2), which is also a downstream target of RANKL mediated signalling [13]. We next checked the activation of ERK1/2 and we observed enhanced phosphorylation of ERK1/2 after 30 min of stimulation with rIL-9 along with MCSF/sRANKL compared to cells stimulated with MCSF/sRANKL (Figure 3b). Similarly, we observed a higher phosphorylation of p38 mitogen-activated protein kinases (P38MAPK) after 30 min of stimulation with rIL-9 along with MCSF/sRANKL compared to cells stimulated with MCSF along with sRANKL (Figure 3c). Furthermore, we investigated the effect of ERK1/2 and P38MAPK inhibition on IL-9 induced osteoclast formation. Inhibition of ERK1/2 enhanced osteoclast formation in both MCSF/sRANKL and MCSF/sRANKL/rIL-9 stimulated conditions. In contrast, P38MAPK inhibition reduced osteoclast formation in both conditions (Figure S2).

Rel-B and p65 play unique roles in osteoclastogenesis [19, 20], therefore, we next checked the impact of IL-9 on them (Figure 3d). We observed higher activation of both p65 and Rel-B in presence of IL-9 stimulation along with MCSF/sRANKL after 12 h as compared to cells stimulated with MCSF and sRANKL. As Rel-B activation is known to induce the expression of peroxisome proliferator-activated receptor gamma coactivator 1-beta (PGC-1β) leading to mitochondrial biogenesis linked to osteoclastogenesis [21], we evaluated the effect of IL-9 on the mRNA expression of PGC-1β and mitochondrial copy number. We observed enhanced expression of *Pgc1b* (Figure 3e) and mitochondrial copy number (Figure 3f) with IL-9 stimulation in RANKL mediated osteoclastogenesis. This observation clearly supports the hypothesis that IL-9 mediated enhanced mitochondrial biogenesis is responsible for the increased osteoclastogenesis.

### IL-9 increases the bone resorptive function of osteoclast

**Figure 4:**
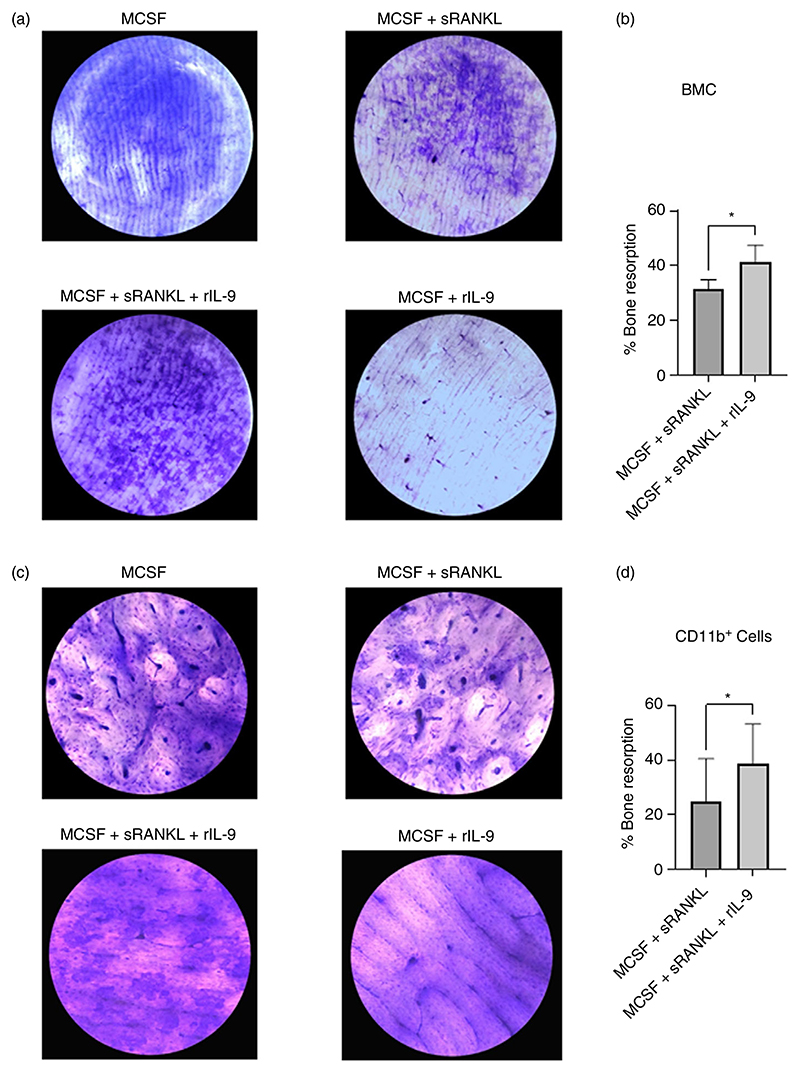
Effect of IL-9 on bone resorption potential of RANKL induced osteoclasts. (a, b) 2 × 10^5^ bone marrow cells (BMCs) were treated as indicated with macrophage colony-stimulating factor (MCSF; 30 ng/mL), soluble receptor activator of nuclear factor κB ligand (sRANKL; 10 ng/mL) or rIL-9 (50 ng/mL) for 15 days as described in the methods. (a) Representative photographs of resorption pits induced by MCSF + sRANKL in presence or absence of rIL-9. Cells stimulated with either MCSF or in combination with rIL-9 did not show any resorption pits. (b) Graph showing the % of bone resorption that was calculated from the total pit area and bone area (*n* = 3). (c, d) 2 × 10^5^ sorted CD11b^+^ cells were treated as indicated with macrophage colony-stimulating factor (MCSF; 30 ng/mL), soluble receptor activator of nuclear factor κB ligand (sRANKL; 10 ng/mL) or IL-9 (50 ng/mL) for 15 days. (c) Representative photographs of resorption pits. (d) Graph showing the % of bone resorption that was calculated from the total pit area and bone area (*n* = 3). Statistical analysis was performed using paired Student’s *t*-test comparing MCSF/sRANKL/rIL-9-treated cells to MCSF/sRANKL treated cells (*p ≤ 0.05).

As we observed enhanced osteoclast formation with rIL-9 stimulation along with MCSF/sRANKL, we next checked the impact of IL-9 on the bone resorptive function of the osteoclasts. We checked the bone resorption of osteoclast derived from BMCs (a heterogeneous cell population) and monocytes (a homogenous cell population). We observed that rIL-9 stimulation along with MCSF/sRANKL enhanced the bone resorption as compared to the MCSF/sRANKL stimulated cells (Figure 4a,b). Similar observations were made with IL-9 treatment along with MCSF/sRANKL in CD11b^+^ cells (Figure 4c,d). Thus, our observation clearly indicates that IL-9 directly enhances the process of osteoclast formation and its bone resorptive function.

### Differential effect of IL-9 on effector and regulatory T cells

**Figure 5:**
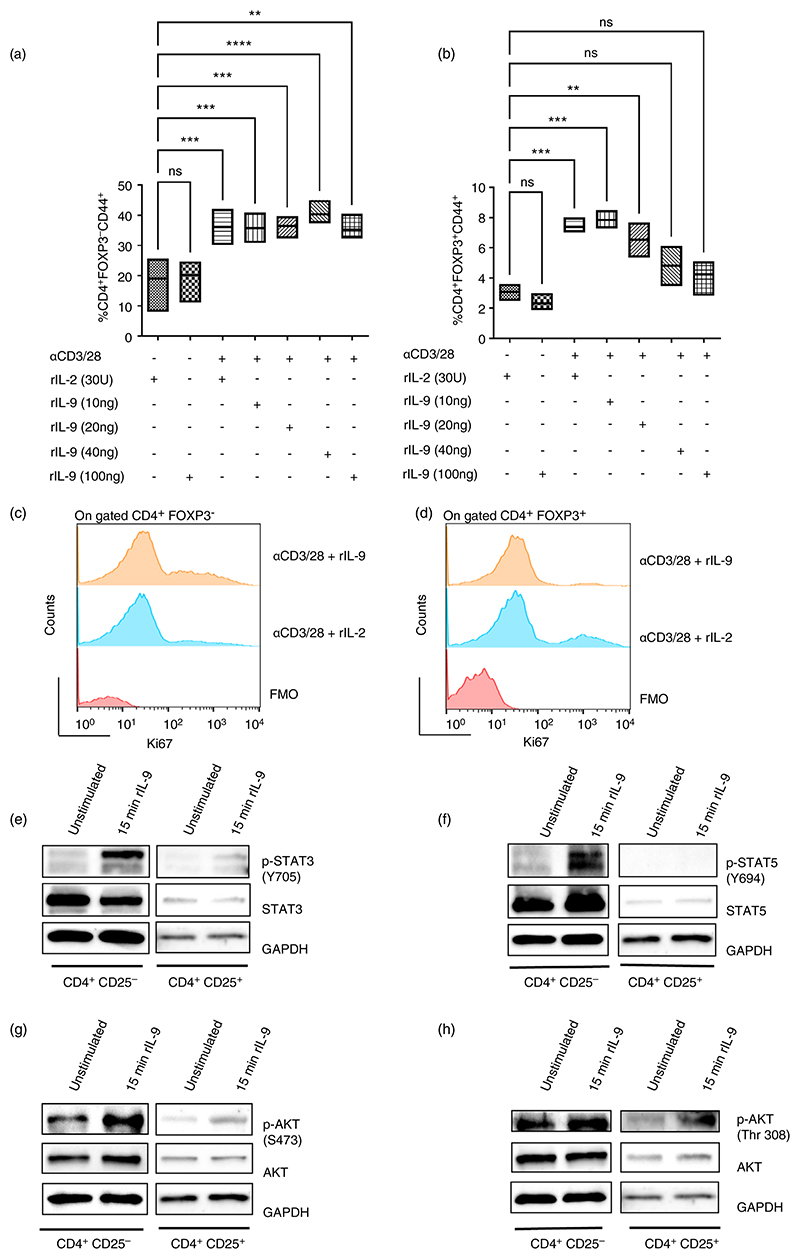
Effect of IL-9 on the functions of T cells. (a, b) Splenocytes were stimulated with anti-CD3/CD28 conjugated dynabeads for 20 h in presence or absence of different concentrations of IL-9 or IL-2 (30 U). Lymphocytes were gated on the basis of their scatter profile (FSC/SSC) followed by gating CD4^+^ cells. On gated CD4^+^ cells, FOXP3^−^ CD44^+^ (activated T effector cells) and FOXP3^+^ CD44^+^ (activated T regulatory cells) were analysed. Cumulative plots showing frequency of CD4^+^FOXP3^−^CD44^+^ (a) and CD4^+^FOXP3^+^ CD44^+^ (b). Statistical analysis was performed using paired one-way ANOVA for multiple comparisons (*n* = 4, **p < 0.005; ***p < 0.0005; ****p ≤ 0.0001). (c, d) Overlay plots showing proliferation (Ki67) of CD4^+^FOXP3^−^ (c) and CD4^+^FOXP3^+^ (d) cells stimulated with αCD3/28 in presence of rIL-2 or rIL-9 for 96 h. (e–h) Sorted CD4^+^CD25^−^ cells (T effector cells) and CD4^+^CD25^+^ cells (T regulatory cells) were stimulated with rIL-9 for 15 min. (e) The immunoblot was probed with an antibody detecting phosphorylated STAT3 (pSTAT-3); STAT3 and GAPDH (*n* = 3). (f) The immunoblot was probed with an antibody detecting phosphorylated STAT5 (pSTAT5); STAT5 and GAPDH (*n* = 3). (g, h) The immunoblot was probed with an antibody detecting phosphorylated AKT (pAKT); AKT and GAPDH (*n* = 3). The GAPDH blot is the same for figures 5 (e) and (h) as well as (f) and (g) in CD4^+^ CD25^+^ (Treg) cells, as the blots are from the same experiment.

As mentioned earlier, in inflammatory conditions, activated T cells play an important role in driving osteoclastogenesis. IL-9 belongs to the γc family and the members of this family profoundly influence the T cell response; especially IL-2 is critical for the functions of T cell subsets. Therefore, we checked the effect of rIL-9 on the activation of Teff cells (CD4^+^ Foxp3^−^) and Treg cells (CD4^+^ Foxp3^+^) after crosslinking and activation with αCD3/28 beads. We observed that T cell stimulation along with increasing concentrations of IL-9 did not alter the frequency of activated Teff cells (CD4^+^ Foxp3^−^CD44^+^) (Figure 5a). In contrast, the frequency of activated Treg cells (CD4^+^ Foxp3^+^ CD44^+^) gradually decreased with increasing concentrations of rIL-9 (Figure 5b), indicating that higher concentrations of IL-9 negatively impacted Treg cell activation. We next checked the proliferation of Teff and Treg cells with stimulation in presence of IL-9 (100 ng/mL). We used IL-2 as a control, as IL-2 is a growth factor of Teff and Treg cells. We observed that IL-9 enhanced the proliferation of stimulated Teff cells (Figure 5c; Figure S3). In contrast, Treg cell proliferation was compromised with αCD3/28 stimulation in presence of IL-9 as compared to IL-2 (Figure 5d; Figure S3). This suggests that although IL-9 and IL-2 belong to the same cytokine family, they have distinct impacts on Treg and Teff cells. We next checked the IL-9 mediated signalling in Teff and Treg cells. Using cell sorting, we purified Teff (CD4^+^ CD25^−^) and Treg (CD4^+^ CD25^+^) cells and stimulated the cells with rIL-9 for 15 min (Figure S4). We observed that STAT3 is phosphorylated with rIL-9 treatment in both Teff and Treg cells (Figure 5e). However, we observed IL-9 induced phosphorylation of STAT5 in Teff cells, but not in Treg cells (Figure 5f). We next checked the phosphorylation of AKT (Thr 308 and Ser473) with IL-9 stimulation in Teff and Treg cells. We observed IL-9 mediated phosphorylation of AKT (Thr 308 and Ser473) in both Teff and Treg cells. Previously, it has been observed that reduced activation of AKT (Ser473) is required for the immune-suppressive function of Treg cells [22]. Thus, from our study it is evident that IL-9 negatively influences Treg cells by inducing phosphorylation of AKT at Ser473.

### IL-9 compromises the anti-osteoclastogenic function of Treg cells

**Figure 6:**
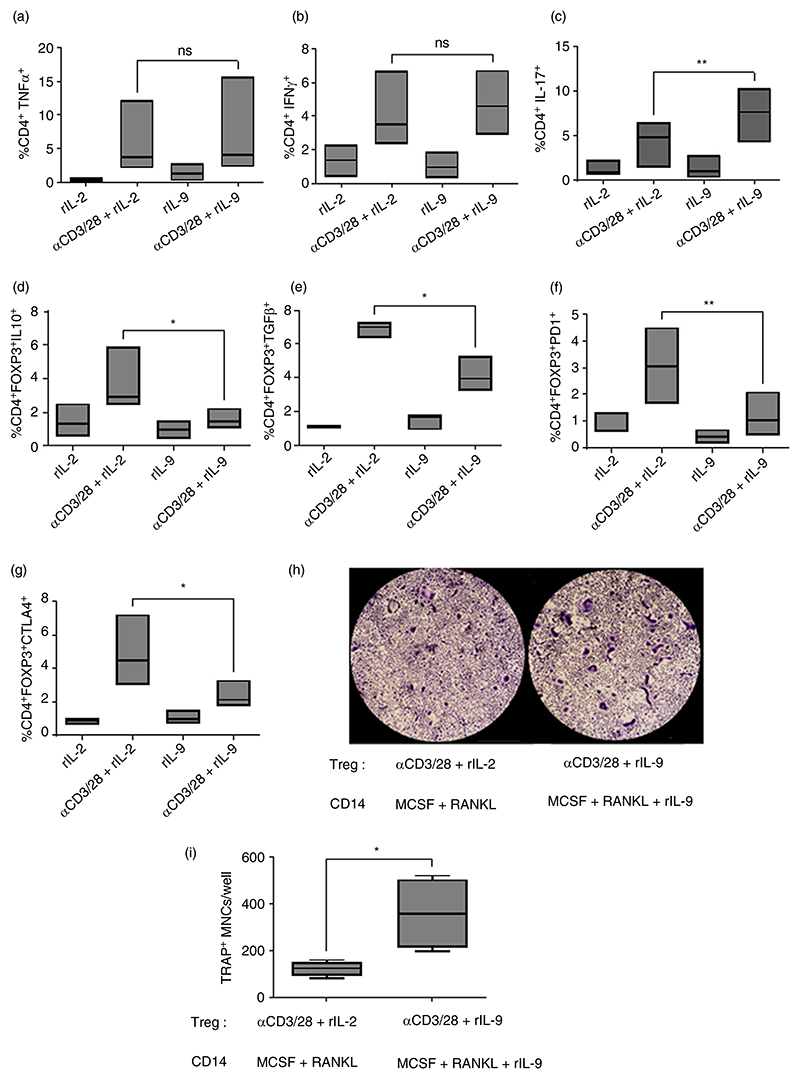
Effect of IL-9 on the functions of T cells. (a–g) Splenocytes were stimulated with anti-CD3/CD28 conjugated dynabeads for 60 h in presence or absence of either IL-9 (100 ng/mL) or IL-2 (30 U). (a–c) lymphocytes were gated on the basis of their scatter profile (FSC/SSC) followed by gating CD4^+^TNFα^+^, CD4^+^IFNγ^+^ and CD4^+^IL-17^+^. Cumulative plots showing frequency of CD4^+^TNFα^+^ (a), CD4^+^IFNγ^+^ (b) and CD4^+^IL-17^+^ (c). (d–g) First, lymphocytes were gated on the basis of their scatter profile (FSC/SSC). On gated CD4^+^ cells, FOXP3^+^ cells expressing IL-10 (d), TGFβ (e), PD1 (f) and CTLA4 (g) were analysed. Cumulative plots showing frequency of CD4^+^FOXP3^+^IL10^+^ (d), CD4^+^FOXP3^+^TGFβ^+^ (e), CD4^+^FOXP3^+^PD1^+^ (f), CD4^+^FOXP3^+^CTLA4^+^ (g). Statistical analysis was performed using paired Student’s *t*-test (*n* = 4; *p < 0.05, **p < 0.005). (h, i) As indicated, stimulated CD11b^+^ cells were co-cultured with stimulated Treg cells in 1:1 ratio for 6–7 days. After 72 h intervals, half of the culture medium was replenished with fresh culture medium containing stimulating factors (MCSF, sRANKL and rIL-9). Cells were then fixed and stained for tartrate-resistant acid phosphatase (TRAP). Using a light microscope, multinucleated (≥3 nuclei) TRAP^+^ cells were counted manually. (h) Representative picture of TRAP^+^ multinucleated cells (MNCs). (I) Graph shows TRAP^+^ MNCs (mean ± SD; *n* = 5). Statistical analysis was performed using paired Student’s *t*-test (*p < 0.05).

Next, we checked the effector function of Teff and Treg cells with stimulation in presence of IL-9. We observed that αCD3/28 crosslinking along with IL-9 does not significantly affect the frequency of IFN-γ and TNF-α expressing CD4 cells (Figure 6a,b). However, IL-9 stimulation along with αCD3/CD28 crosslinking significantly enhanced the frequency of IL-17 producing CD4 cells (Figure 6c).

Treg cells can mediate their effect through contact independent actions involving suppressive cytokines (IL-10 and TGF beta) and contact dependent involving immune inhibitory molecules like programmed cell death protein 1 (PD1) and cytotoxic T lymphocyte associated protein −4 (CTLA4). Therefore, we checked the effect of IL-9 on these aspects of Treg cells. We observed that with IL-9 stimulation along with αCD3/CD28, reduced the frequency of Treg cells expressing IL-10, TGF-β, CTLA-4 and PD1 (Figure 6d–g). These observations clearly suggest that IL-9 failed to support the function of Treg cells.

Next, we wanted to check the effect of IL-9 stimulated Teff and Treg cells on osteoclastogenesis. For this purpose, sorted Teff and Treg cells were stimulated as indicated. Then the activated T cells were mixed with MCSF/sRANKL stimulated monocytes treated with or without IL-9. As previously documented, we observed that co-mixing stimulated Teff cells with MCSF/sRANKL stimulated monocytes inhibited the process of osteoclastogenesis [23]. This is due to the secretion of IFN-γ from activated T cells, which is known to inhibit osteoclastogenesis.

We next looked for the effect of co-mixing stimulated Treg cells with stimulated monocytes. Zaiss et al. have nicely shown that Treg cells inhibit the process of osteoclastogenesis [12]. We also observed inhibition of osteoclastogenesis with Treg cells stimulated with αCD3/CD28 and rIL-2. However, this inhibitory effect of stimulated Treg cells on osteoclastogenesis was lost in presence of IL-9 (Figure 6h,i). This clearly indicates that IL-9 negatively impacts the suppressive functions of Treg cell and thus Treg cells were unable to inhibit osteoclastogenesis in presence of IL-9.

## Discussion

IL-9 has been studied in great depth in the T cell mediated immune response in various pathological conditions ranging from asthma, autoimmunity to cancer. This might be due to the fact that it belongs to the family of γ chain receptor which includes cytokines (IL-2, IL-4, IL-7, IL-15 and IL-21) that are critical players in adaptive immune response as evident from the mutations observed in γ chain receptor causing severe combined immunodeficiency (SCID) diseases [24]. In pathological conditions, depending on the cellular context, IL-9 can either participate in mediating inflammation or resolution, which make its biology very interesting [25, 26]. Recently, we showed in RA that IL-9 enhances osteoclast differentiation and function indicating a role in influencing bone remodelling in chronic inflammatory conditions [17]. Here, we observed that IL-9 enhances the activation of STAT3, P38MAPK and ERK1/2 in the process of osteoclastogenesis. Activation of P38MAPK is known to positively regulate osteoclast differentiation and its function [27]. Indeed, we also observed reduction in osteoclast formation in presence of P38MAPK inhibitor. In contrast, the effect of ERK1/2 activation in osteoclast formation is not clear; a recent report showed that ERK inhibition enhanced osteoclast formation in RAW264.7 cells by down regulating the expression of negative regulators of osteoclast formation [28]. We also observed similar findings in BMCs with ERK inhibitor. However, detailed studies are required to understand the intricate mechanism. The importance of STAT3 in osteoclastogenesis was highlighted with the recent observation that osteoclast-specific STAT3 deficiency in mice enhanced bone mass due to impairment of osteoclast formation and function [18]. The authors showed that STAT3 activation is indispensible for mediating osteoclastogenesis due to its role in regulating NFATc1 transcription [18]. We also observed that STAT3 inhibitor treated cells failed to show any osteoclast formation either with MCSF/sRANKL or in the presence of IL-9. Thus, it appears that IL-9 mediated STAT3 activation is instrumental in promoting RANKL induced osteoclastogenesis.

RANKL mediated osteoclastogenesis is known to activate both classical and alternative NFkB pathways. Interestingly, we also observed that IL-9 stimulation augments the RANKL induced activation of NFkB and Rel-B. Rel-B activation links mitochondrial biogenesis to osteoclastogenesis through modulating the expression of PGC-1β [21]. Indeed, we also observed that IL-9 stimulation in RANKL induced osteoclastogenesis enhanced the transcription of PGC-1β leading to accelerated mitochondrial biogenesis. Our observation hints that IL-9 influences the osteoclast formation and function through augmenting mitochondrial biogenesis rather than an increase in the induction of OC genes.

The observed effect of IL-9 on osteoclastogenesis is also shared by its family members. IL-2 has been shown to enhance the resorptive potential of osteoclasts [29, 30]. There are ambiguous observations regarding the impact of IL-7 on osteoclasts and requires thorough investigation [31–33]. Similar to IL-9, IL-15 and IL-21 were observed to promote RANKL induced osteoclastogenesis [34, 35]. Additionally, IL-21 was shown to induce osteoclastogenesis in a RANKL independent manner in RAW-264.7 cells [36]. In this study, we also checked the effect of IL-9 on RANKL induced osteoclastogenesis in RAW-264.7. However, we did not observe any difference between RANKL induced osteoclastogenesis in presence or absence of IL-9 in RAW-264.7 (Figure S5a,b). We found that this was due to the low expression of IL-9R on RAW-264.7 cells (Figure S5c). Thus, RAW 264.7 cells are not the ideal cell type for studying IL-9 mediated signalling events.

IL-9 has been shown to promote inflammation in various inflammatory diseases [37–41]. Here, we also found that higher concentrations of IL-9 enhanced the activation and proliferation of Teff cells. In addition, we showed that high concentrations of IL-9 enhanced the frequency of IL-17 producing T cells, which is a known player in mediating inflammatory bone disorders. This observation is in line with previous observations on chronic inflammatory diseases like MS and RA, where IL-9 has been reported to promote Th17 cells [39, 42]. We showed that IL-9 signals through STAT3, STAT5 and AKT in Teff cells. Mathur et al. have shown that STAT3 activation is critical for T cell mediated production of IL-17 [43]. This report suggests that IL-9 possibly promotes IL-17 producing T cells primarily through STAT3 activation. Whereas, IL-9 mediated phosphorylation of STAT5 and AKT in Teff cells might possibly help in mediating its effect on cell proliferation, survival and differentiation [44, 45]. Contrary to its positive impact on Teff cells, we showed that IL-9 does not support the proliferation, secretion of suppressive cytokines (IL-10 and TGF-β) and expression of co-inhibitory molecules (PD1 and CTLA4) critical for mediating the immuno-suppressive function of Treg cells. The effect of IL-9 mediated impairment of Treg cell functions on osteoclastogenesis was tested using co-culturing Treg cells with osteoclast precursors. We showed that stimulated Treg cells in the presence of IL-9 were unable to inhibit osteoclast formation. The negative impact of IL-9 on Treg cells is possibly due to the activation of AKT at Ser 473 in Treg cells. Defect in the phosphorylation of AKT at Ser 473 in Treg cells is responsible for mediating its immuno-suppressive functions [22]. In addition, IL-9 failed to activate STAT5, which is crucial for Treg function and maintenance of FOXP3. Previously, it was shown that Tregs expressing a constitutively active form of STAT5 were superior in mediating immune suppression and in protecting mice from graft-versus-host disease [46]. Thus, our study provides a possible mechanism by which IL-9 can impair the functions of Treg cell during inflammatory bone disease leading to excessive inflammation and osteoclastogenesis.

Together, our study suggests that IL-9 can influence osteoclast formation and its function in chronic inflammatory condition through three ways (i) by directly enhancing osteoclast formation and function from its precursor cells, (ii) by enhancing the frequency of osteoclastogenic IL-17 producing T cells and (iii) by inhibiting anti-osteoclastogenic Treg cells.

## Supplementary Material

Data S1

Supplementary figures

Supplementary tables

## Data Availability

The data that support the findings of this study are available in the manuscript and can be obtained from the corresponding author upon request.
