# Supplementary material for: Mechanistic insight of interleukin-9 induced osteoclastogenesis
Source: Immunology. Author manuscript; Available in PMC 2024 May 26. (PMC7615986; doi:10.1111/imm.13630)
Supplement: Data S1 [file EMS196185-supplement-Data_S1.docx]

**Supplementary Methods**

**Isolation of bone marrow cells, monocytes and bone marrow-derived macrophages**

Bone marrow cells (BMCs) were isolated from the femur, tibia and humerus of 6–12 weeks old C57BL/6 mice. Isolated cells were treated with red blood cell lysis buffer (RBC) and centrifuged (1300 rpm, 5 mins). Cells were cultured overnight in α- Minimal Essential Medium (MEM) supplemented with glutamine (anprotec, Bruckberg, Germany), 10% heat-inactivated foetal calf serum (FCS; Biochrom GmbH, Berlin, Germany), 1% Penicillin and Streptomycin (anprotec, Bruckberg, Germany). Next day, the non-adherent BMCs were used for further experiments.

Monocytes were isolated from mouse bone marrow cells using EasySep™ Mouse Monocyte isolation kit as per the recommendation of manufacture’s protocol (STEMCELL Technologies, Vancouver, Canada,).

For generating bone marrow derived macrophages (BMDM), BMCs were cultured using L929-cell conditioned medium (LCCM) for 6 days as mentioned previously ^7^. BMDM cells were cultured in Dulbecco's Modified Eagle Medium (DMEM; Anprotec, Bruckberg, Germany) supplemented with 10% FCS and 1% Penicillin and Streptomycin at humidified atmosphere (95%), 5% CO_2_ and 37°C.

**RAW264.7**

RAW264.7 cells (ATCC) were cultured in DMEM, supplemented with 10 % heat-inactivated FCS, 1% Penicillin and Streptomycin. The cells were passaged every 2–3 days in a ratio of 1:20.

**Isolation of splenocytes and T cells**

Spleen was collected from C57BL/6 mice and was manually disrupted in PBS containing 2% FCS. The debris and aggregates were removed by passing the cell suspension through a 45 µm cell strainer. Cells were centrifuged at 1300 rpm for 10 mins. The cell pellet was treated with RBC lysis buffer followed by centrifugation. Splenocytes were cultured in complete RPMI 1640 media for subsequent experiments.

CD4^+^ CD25^-^ (T effector) and CD4^+^ CD25^+^ (T regulatory) cells were isolated from spleen using EasySep™ Mouse CD4+CD25+ Regulatory T Cell Isolation Kit II (STEMCELL Technologies, Vancouver, Canada).

**RNA isolation, cDNA preparation and Real time quantitative polymerase chain reaction**

RNA was isolated from cells using innuPREP RNA Mini Kit 2.0 (Analytik Jena AG, Jena, Germany) and quantified by using Nanodrop. 0.5 µg - 1 µg of RNA in 12 µL RNase-free H_2_O were used as input RNA for cDNA synthesis with biotechrabbit GmbH cDNA synthesis kit (Berlin, Germany). Real-time PCR analysis was performed using 2x qPCRBIO SyGreen Mix Hi-ROX (PCR Biosystems Ltd, London, UK) on the StepOnePlus Real-Time PCR System (ThermoFischer Scientific, Massachusetts, United States). Sequence of primer pairs used are listed in the **Supplementary Table 1**. Relative gene expression of target genes was calculated and Rsp29 was used as house-keeping gene of using 2-[Ct(target gene)−Ct(reference gene)].

**TRAP Assay**

2.5 × 10^5^ cells were plated in 1 ml of complete medium in 24-well plates and treated as described in figure legends with MCSF and sRANKL (bio-techne, Minneapolis, USA) in presence or absence of IL-9 (PeproTech, NJ, USA). Cells were then fixed and stained using Acid Phosphatase, Leukocyte (TRAP) Kit (Sigma, St. Louis, MO, USA). TRAP-positive multi-nucleated cells were scored as osteoclasts.

For checking the effect of inhibitors on TRAP positive multi nucleated cells, we used 1 × 10^6^ BMCs. Cells were treated with 7.5 µM STAT3 Inhibitor XIII C188-9 (Sigma, Taufkirchen, Germany), 15 µM ERK Inhibitor CAS 1049738-54-6 (Sigma, Taufkirchen, Germany) and 0.2 µM p38 MAP Kinase Inhibitor IV CAS 1638-41-1 (Sigma, Taufkirchen, Germany) prior to stimulation as indicated.

**Bone resorption assay**

2 × 10^5^ cells were plated in 1 ml of complete medium in 24-well plates and stimulated as mentioned. On day 3, cells were transferred to a 96-well plate containing bovine cortical bone slices (http://Boneslices.com, Jelling, Denmark) and cultivated for 12 days. For measurement of resorbed bone, bone slices were washed with thoroughly with water, and stained with 0.1% toluidine blue (Sigma-Aldrich, Missouri, USA). The pits developed a blue to purple colour. Resorption pits were visualized using light microscopy Rebel Microscope (ECHO A Bico Company, CA, USA). Resorption pits were further analysed with FIJI software.

**Flow Cytometry (FC)**

For surface staining, 0.5 x 10^6^ cells were washed with staining buffer (phosphate-buffered saline, 2% FBS) and stained using antibodies listed in the **Supplementary Table 2** for 30 minutes at 4˚C. For intracellular staining, surface-stained cells were then fixed and permeabilized using buffer set (eBioscience™ FOXP3 / Transcription Factor Staining Buffer Set; San Diego, CA, USA). Cells were then stained for intracellular molecules for 30 minutes in the dark. Data was acquired using BD FACS Canto (BD Biosciences, San Jose, CA, USA). Data analysis was done using Flow Jo software (Tree Star, Inc., Ashland, OR, USA).

**Immunoblotting**

A total of 1 × 10^6^ cells were stimulated as indicated. Stimulated cells were lysed using a lysis buffer (1% NP-40, 0.25% deoxycholate, 50 mM Tris pH7.4, 150 mM NaCl, 1 mM EDTA, 1 mM Na3VO4) supplemented with a Phosphatase and Protease-Inhibitor Cocktail. Equal amounts of protein were loaded to ProGel Tris Glycine 4-20% polyacrylamide gel, and then transferred to the nitrocellulose blotting membrane via semi-dry blot system. Membranes were blocked in 1x blocking buffer for 30 minutes at room temperature and incubated with the specific primary antibody as listed in **Supplementary Table 2** for overnight at 4°C. Probed bands were detected by the appropriate HRP-conjugated secondary antibody (Cell Signaling, Massachusetts, USA) upon incubation for 1 hour at room temperature. Membranes were developed using the ECL substrate WESTAR 𝜂C Ultra 2.0 (Cyanagen; Bologna, Italy) and visualized on ChemoStar image (Intas Science Imaging).
