# Supplementary material for: Mechanistic insight of interleukin-9 induced osteoclastogenesis
Source: Immunology. Author manuscript; Available in PMC 2024 May 26. (PMC7615986; doi:10.1111/imm.13630)
Supplement: Supplementary figures [file EMS196185-supplement-Supplementary_figures.docx]

**Supplementary Figures and their Legends**

**
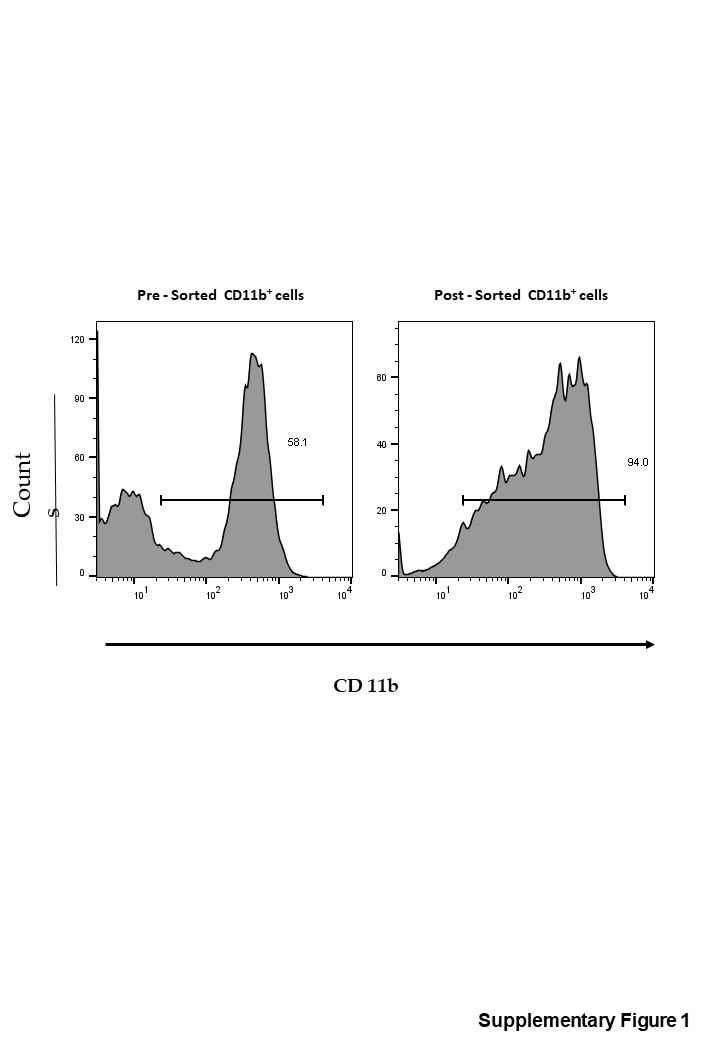
**

**Figure1. Sorting of monocytes from bone marrow cells.** Monocytes were sorted from bone marrow cells using EasySep™ Mouse Monocyte isolation kit. Histogram showing the CD11b positive cells in pre-sorted cells and post-sorted cells.


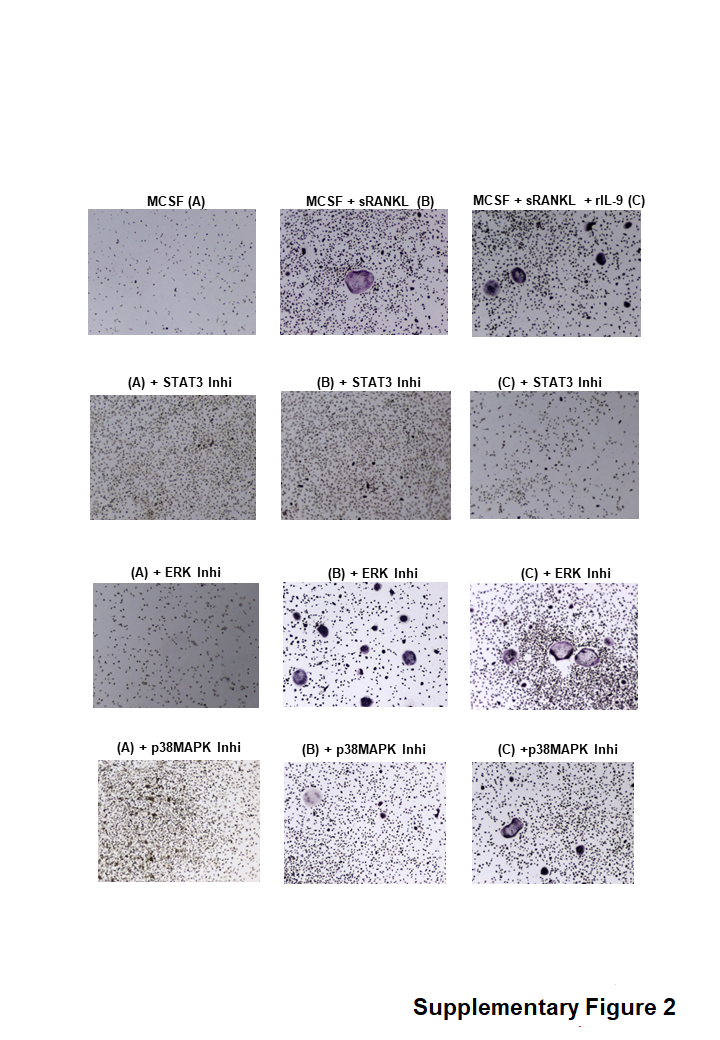


**Figure 2: Effect of inhibiting STAT3, ERK1/2 and p38MAPK on IL-9 induced osteoclast formation.**

1x10^6^ bone marrow cells (BMCs) were pre-treated as indicated with STAT3 inhibitor (7.5 µM), ERK1/2 Inhibitor (15 µM) and p38MAPK inhibitor (0.2 µM) for 60 minutes. Cells were then stimulated with macrophage colony-stimulating factor (MCSF; 30 ng/mL), soluble receptor activator of nuclear factor κB ligand (sRANKL; 10 ng/mL), or IL-9 (50 ng/mL) for 5-6 days. After 72 hours intervals, half of the culture medium was replenished with fresh culture medium containing stimulating factors (MCSF, sRANKL, and rIL-9). Prior to restimulation, cells were treated with inhibitors. Cells were then fixed and stained for tartrate-resistant acid phosphatase (TRAP). Representative picture of TRAP^+^ cells (N=2).


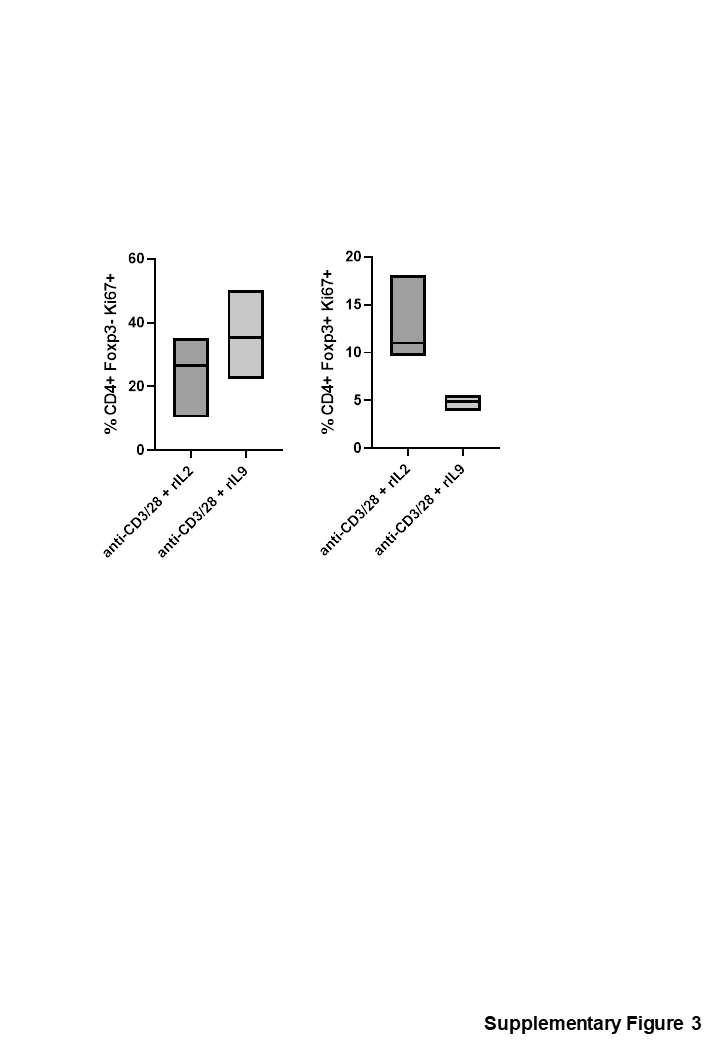


**Figure 3. IL-9 mediated effect on proliferation of T effector and T regulatory cells.**

Cumulative Plots showing frequency of CD4+FOXP3- Ki67+(Left) and CD4+FOXP3+ Ki67+(Right).

**
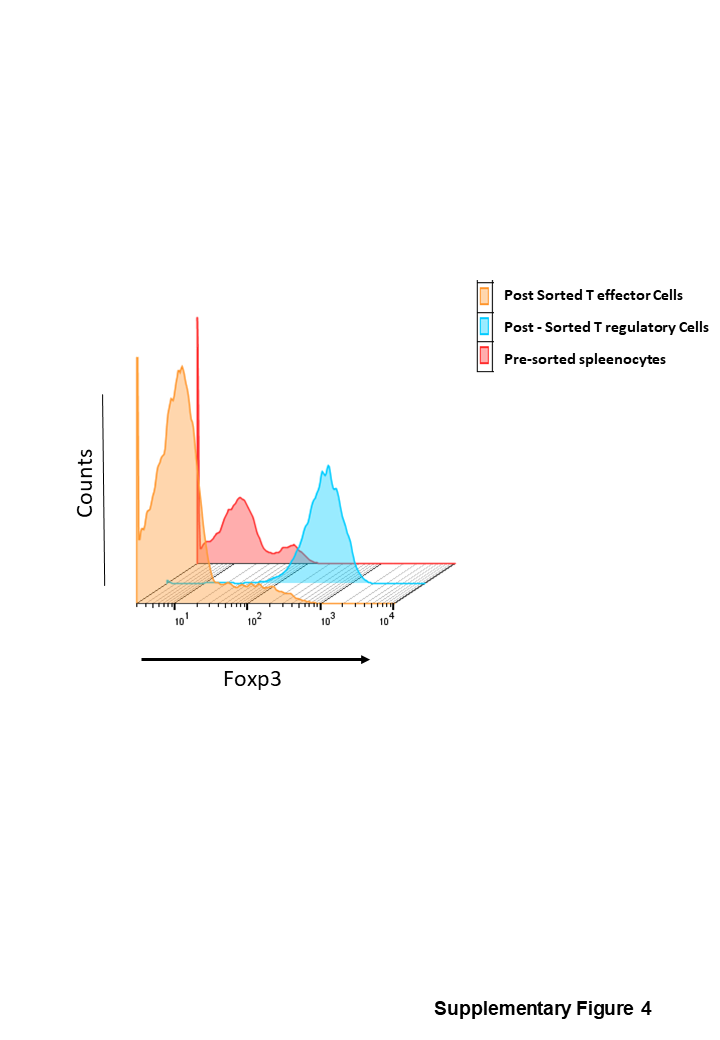
**

**Figure 4. Sorting of T effector and T regulatory cell from spleen.** CD4+ Foxp3- (T effector) and CD4^+^ Foxp3^+^ (Regulatory T cells) were sorted from splenocytes using EasySep™ Mouse CD4^+^CD25^+^ Regulatory T Cell Isolation Kit II. Overlay showing Foxp3+ in pre-sorted cells, post-sorted T effector and T regulatory cells.

**
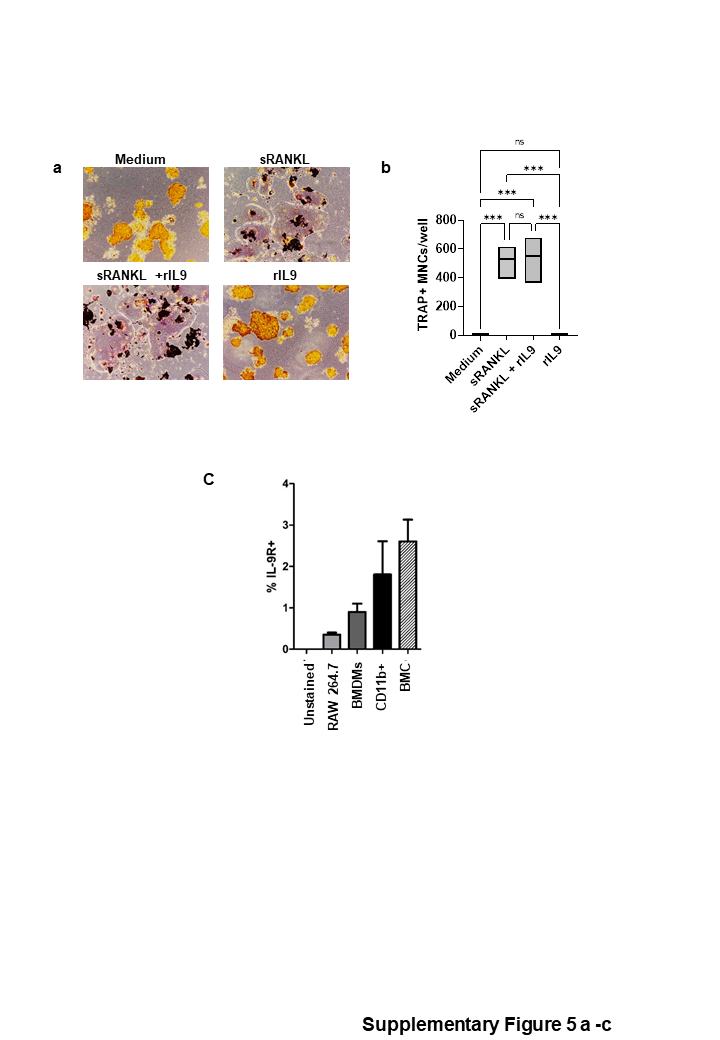
**

**Figure 5 Effect of IL-9 on osteoclast formation in RAW 264.7 macrophages:**

5 x 10^3^ RAW264.7 macrophages were treated as indicated with soluble receptor activator of nuclear factor κB ligand (sRANKL; 50 ng/mL), or IL-9 (100 ng/mL) for 4 days. After 48-hour cells, half of the culture medium was replenished with fresh culture medium containing stimulating factors (sRANKL, and rIL-9). Cells were then fixed and stained for tartrate-resistant acid phosphatase (TRAP). Using a light microscope, multinucleated (≥3 nuclei) TRAP^+^ cells were counted manually. (**a**) Representative picture of TRAP+ multinucleated cells (MNCs). (**b**) Graph shows TRAP^+^ MNCs (mean ± SD; n = 3). Statistical analysis was performed using one- way Anova for multiple comparison (*: p  ≤  0.05; ***: p  ≤  0.0005). (**c**) Expression of the IL-9R in RAW264.7, Bone marrow derived macrophages (BMDM), bone marrow cells (BMCs) and CD11b^+^ cells (n=2). Cells were stained with PE anti-mouse IL-9R antibodies. Graph showing frequency of IL-9R positive cells in different cell types.
