# Supplementary material for: Mechanistic insight of interleukin-9 induced osteoclastogenesis
Source: Immunology. Author manuscript; Available in PMC 2024 May 26. (PMC7615986; doi:10.1111/imm.13630)
Supplement: Supplementary tables [file EMS196185-supplement-Supplementary_tables.docx]

**Supplementary Table 1: List of Primers used in the study**

| **Primer** | **Sequence 5’→3’** |
| --- | --- |
| *Rsp29*, Mouse Forward primer | AGCCGACTCGTTCCTTTCTC |
| *Rsp29*, Mouse Reverse primer | CGTATTTGCGGATCAGACC |
| *Pgc1b*, Mouse Forward primer | CTCCAGGCAGGTTCAACCC |
| *Pgc1b*, Mouse Reverse primer | GGGCCAGAAGTTCCCTTAGG |
| *Ctsk*, Mouse Forward primer | AGGGAAGCAAGCACTGGATA |
| *Ctsk*, Mouse Reverse primer | GCTGGCTGGAATCACATCTT |
| *Acp5*, Mouse Forward primer | TTCCAGGAGACCTTTGAGGA |
| *Acp5*, Mouse Reverse primer | GGTAGTAAGGGCTGGGGAAG |
| *Dcstamp*, Mouse Forward primer | AAAACCCTTGGGCTGTTCTT |
| *Dcstamp*, Mouse Reverse primer | GTTCCTTGCTTCTCTCCACG |
| *Nfatc1*, mouse Forward primer | CAGGGCTCACTATGAGACGG |
| *Nfatc1*, mouse Reverse primer | AGCTGTAGCGTGAGAGGT |
| *Ocstamp,* mouse Forward primer | TGGGCCTCCATATGACCTCGAGTAG |
| *Ocstamp,* mouse Reverse primer | TCAAAGGCTTGTAAATTGGAGGAGT |
| *Atp6v0d2*, mouse Forward primer | GTGCCAAATGAGTTCAGAGTGATG |
| *Atp6v0d2*, mouse Forward primer | TCAGATCTCTTCAAGGCTGTGCTG |
| mtDNA, Mouse Forward primer | CTAGAAACCCCGAAACCAAA |
| mtDNA, Mouse Reverse primer | CCAGCTATCACCAAGCTCGT |
| nucDNA B2M, Mouse Forward primer | atgggaagccgaacatactg |
| nucDNA B2M, Mouse Reverse primer | cagtctcagtgggggtgaat |

**Supplementary Table 2. List of antibodies used in the study and its specifications**

| **Antibodies** | **Company** | **Identifier** |
| --- | --- | --- |
| Mouse monoclonal anti-GAPDH | Proteintech | 60004-1-Ig |
| Rabbit monoclonal anti-pAkt (Thr308) | Cell Signaling Technology (CST) | 2965 |
| Phospho-Akt (Ser473) (D9E) XP® Rabbit mAb | CST | 4060 |
| Rabbit monoclonal anti-Relb | CST | 4922 |
| Rabbit monoclonal anti-pNF-κB p65 (Ser536) | CST | 3033 |
| Rabbit monoclonal anti-pStat3 (Tyr705) | CST | 9145 |
| Rabbit monoclonal anti-STAT3 | CST | 4904 |
| Rabbit polyclonal anti-Cytochrome c | Proteintech | 10993-1-AP |
| Goat monoclonal anti-mouse IgG, HRP | CST | 7076 |
| Goat monoclonal anti-Rabbit IgG, HRP | CST | 7074 |
| STAT5 | Santa Cruz Biotechnology | SC-835 |
| Phospho-STAT5 (Y694) | CST | 9351 |
| FITC anti mouse CD4 (Clone: Gk1.5) | Biolegend | 100406 |
| PE/CY7 anti mouse IL10 (Clone: JES5-16E3) | Biolegend | 505025 |
| PE anti mouse FOXP3 (Clone:MF14) | Biolegend | 126404 |
| PE/CY5 anti mouse CD4 (Clone: GK1.5) | Biolegend | 100410 |
| PE/CY7 anti mouse KI67 (16A8) | Biolegend | 652425 |
| FITC anti mouse LAP (TGF BETA) (clone: TW7-16B4) | Biolegend | 141413 |
| FITC anti mouse CD279 (PD-1) | Milteni Biotech | 130-111-952 |
| APC anti mouse/human CD44 | Biolegend | 102011 |
| APC anti mouse TNF-α | Milteni Biotech | 130-123-277 |
| APC anti mouse IFN-γ | Milteni Biotech | 130-123-283 |
| PE/CY7 anti mouse CD152 (UC10-4B9) | Biolegend | 106313 |
| PE/CY7 anti mouse IL17A (TC11-18H10.1) | Biolegend | 506921 |
| APC anti-mouse CD25 Antibody (PC61) | Biolegend | 102011 |
| APC anti – mouse CD4 Antibody (RM4-5) | Proteintech | APC-65141 |
| PE anti-mouse CD129 (IL-9R) Antibody | Biolegend | 158803 |
| FITC anti-mouse/human CD11b Antibody | Biolegend | 101205 |
